# Moonlighting at the Poles: Non-Canonical Functions of Centrosomes

**DOI:** 10.3389/fcell.2022.930355

**Published:** 2022-07-14

**Authors:** Laurence Langlois-Lemay, Damien D’Amours

**Affiliations:** Department of Cellular and Molecular Medicine, Ottawa Institute of Systems Biology, University of Ottawa, Ottawa, ON, Canada

**Keywords:** centrosomes, spindle pole bodies, MTOCs, Cdc5, PLK1, cell cycle

## Abstract

Centrosomes are best known as the microtubule organizing centers (MTOCs) of eukaryotic cells. In addition to their classic role in chromosome segregation, centrosomes play diverse roles unrelated to their MTOC activity during cell proliferation and quiescence. Metazoan centrosomes and their functional doppelgängers from lower eukaryotes, the spindle pole bodies (SPBs), act as important structural platforms that orchestrate signaling events essential for cell cycle progression, cellular responses to DNA damage, sensory reception and cell homeostasis. Here, we provide a critical overview of the unconventional and often overlooked roles of centrosomes/SPBs in the life cycle of eukaryotic cells.

## 1 Introduction

Ever since the centrosome was first discovered in the late 1800s, intense research efforts have been devoted to understanding its roles and life cycle in eukaryotic organisms. In their classic roles as microtubule-organizing centers (MTOCs), centrosomes and SPBs are classified amongst the most primitive organelles but gained complex ancillary functions throughout evolution (Bornens and Azimzadeh, 2007; Nabais et al., 2020). Increasingly, centrosomes are now recognized as important determinants of cell differentiation, self-renewal and aging processes in multicellular organisms.

Visualized for the very first time through electron microscopy, SPBs were described as “small knobs” found at either ends of a long straight fiber during mitosis (Robinow and Marak, 1966). Subsequent studies uncovered that SPBs and centrosomes are morphologically distinct; SPBs are tri-layer structures closely embedded in the nuclear membrane whereas centrosomes are surrounded by pericentriolar material. However, both function as MTOCs. Interestingly, a third class of eukaryotic organelle, the nucleus-associated bodies (NABs), is typically responsible for MTOC-related functions in amoebozoans (Gräf et al., 2015; Gräf, 2018; Ito and Bettencourt-Dias, 2018).

Beyond MTOC activities, centrosomes/SPBs also promote cell signaling events induced by diverse stimulatory and stress signals. Here, we will review the non-canonical roles of MTOCs in cell homeostasis, with a specific focus on how the structural organization and subcellular position of centrosomes/SPBs play a central role in the modulation of cellular processes.

### 1.1 Function and Structural Organization of Eukaryotic MTOCs: An Overview

#### 1.1.1 Centrosomes as MTOCs

**FIGURE 1:**
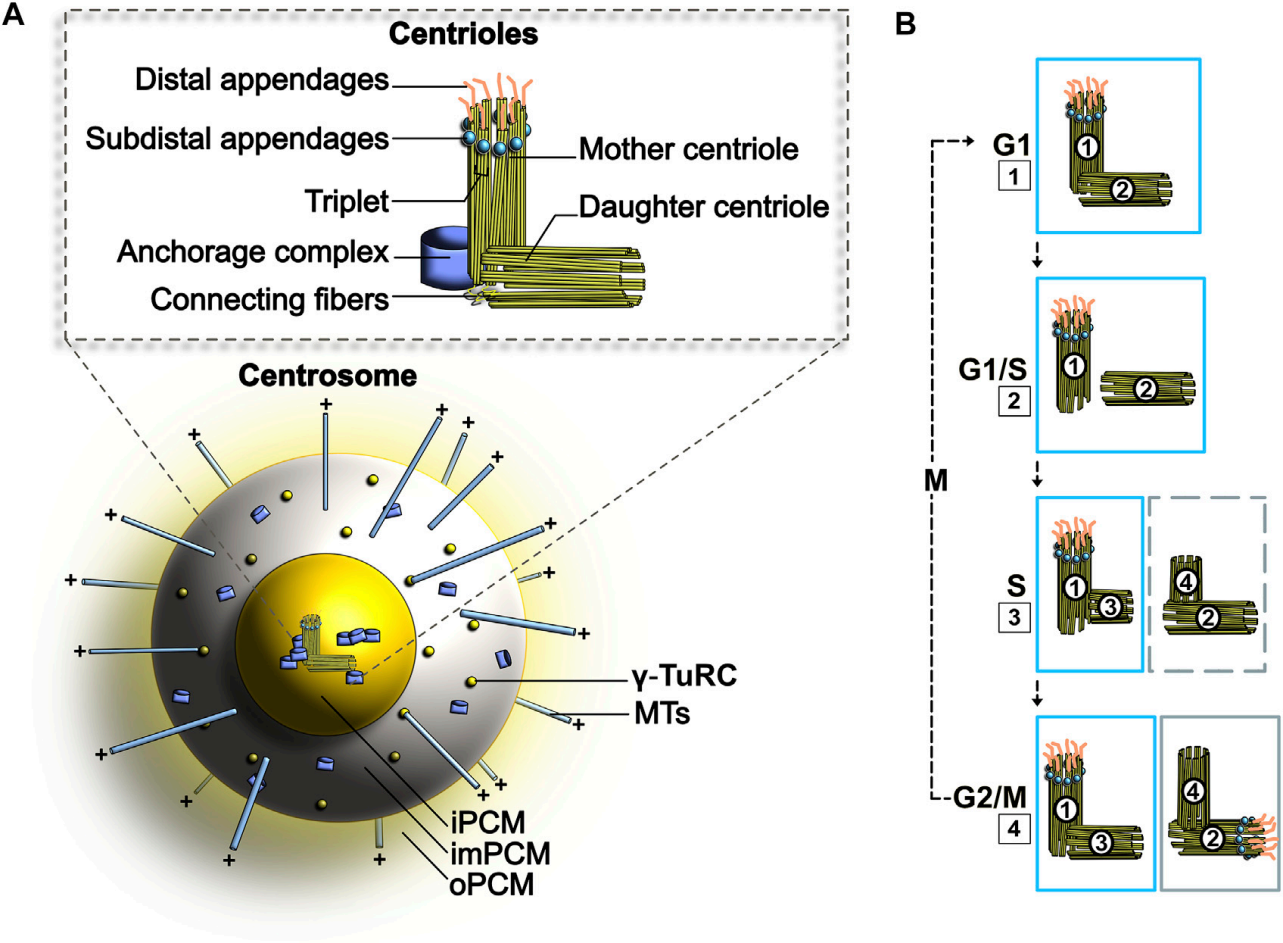
**(A)** Schematic representation of the centrosome. PCM, Pericentriolar material; iPCM, Inner PCM; imPCM, Intermediate PCM; oPCM, Outer PCM **(B)** Centrosome duplication cycle. The duplication of centrosomes is termed semi-conservative, as each older centriole will generate a new centriole. **1–2**. At the G1/S transition, the two centrioles separate. **3**. In S phase, PCM forms around each parting centriole. **4.** Daughter centrioles expand orthogonally and reach opposite poles. See text for more details.

Characterized as a protein-dense scaffolding structure responsible for the nucleation of *α*- and *β*-tubulin, centrosomes arrange and anchor microtubules that form the bipolar spindle in mitosis (reviewed in Wu and Akhmanova, 2017; Gomes Pereira et al., 2021) (Figure 1). The main microtubule nucleator is the γ-tubulin ring complex (γ-TuRC), a highly conserved complex responsible for the capping of microtubule minus ends (Oakley et al., 1990; Zheng et al., 1995). Formed of several proteins including γ-tubulin and actin (Liu et al., 2020; Wieczorek et al., 2020), this complex is located in the pericentriolar material (PCM) and was shown to rely on pericentriolar proteins such as CDK5RAP2 to attach to centrosomes (Fong et al., 2008). The γ-TuRC complex, operating as an organizational template for the nucleation of microtubules, forms the cytoplasmic microtubule array in interphase as well as the mitotic spindle during mitosis and was shown to regulate nucleation dynamics via conformational changes (Consolati et al., 2020). From interphase to mitosis, the function of centrosomes as MTOCs is highly dynamic and supports the ongoing division of proliferating cells (Mazia, 1987). Both the size and function of centrosomes as MTOCs may fluctuate according to the state of a given cell, or even its cell type (Decker et al., 2011). To behave in such a dynamic manner, MTOCs rely on centrosomal components and associated proteins that enrich at the centrosomes to stabilize or release microtubule organization and involve a large array of components that can even selectively enrich to one centriole over the other throughout the cell cycle (Andersen et al., 2003; Jakobsen et al., 2011). Combined together, all these factors allow for a personalized MTOC function specifically catered to cell conditions at a given time to accurately support cell cycle progression through microtubule nucleation.

Aside from its classic function as MTOC, the centrosome also plays crucial roles in cell polarity, shape and migration. When Van Beneden first discovered the centriole in 1883 (Van Beneden, 1883), he hypothesized that the polarity of a cell could be conferred by the orientation of both its nucleus and centrosome (Luxton and Gundersen, 2011). The nuclear-centrosomal (NC) axis exists in the majority of metazoan differentiated cell types, as well as in some unicellular organisms including yeast (Nelson, 2003). The polarity of a cell defined by the orientation of its centrosome is an important feature at the core of many biological processes. Research performed on normal fibroblast to study wound healing reported that both the Golgi apparatus and the centrosome (MTOC) were necessary for directional migration towards the edge of a lesion. Authors speculated that the coordinated orientation of both the MTOC and the Golgi apparatus towards the wound was required to modulate vesicular transport to the edge of the cell, thus leading to the growth of this extremity towards the wound (Kupfer et al., 1982). Akin to this, the centrosome was also reported to play a crucial role in directional mesenchymal cell migration. In a study published in 2017, Zhang and others used micropatterned one-dimensional adhesive strips to study cell polarity in mesenchymal cells and reported that the centrosome was involved in directional cell migration. Specifically, the centrosome was proposed to dynamically localize at the rear of mesenchymal cells to organize the microtubule network and distribute signals related to protrusive activity as a way to establish tail formation during directional migration (Zhang and Wang, 2017).

Asymmetric cell division, a process equally reliant on cell polarity for its occurrence, can also depend on the orientation of centrosomes to effectively reach completion (as reviewed in Chen and Yamashita, 2021). Asymmetric cell division is a common process routinely observed from yeast to humans. In *S. cerevisiae*, aging determinants are partitioned asymmetrically, resulting in a young daughter bud expanding from an older parental yeast. This process directly impacts the replicative lifespan of both parental and daughter cells, which represents the finite number of divisions a cell can undertake before reaching senescence (Longo et al., 2012). Spindle orientation and other factors established by the cell polarity machinery can guide this asymmetric process, which results in the transfer of new components such as mitochondria, endoplasmic reticulum (ER), vacuoles and rejuvenating factors to the daughter cell whilst a number of older components remain in the parental cell (Higuchi-Sanabria et al., 2014). Moreover, SPBs themselves undergo asymmetric inheritance. The older parental SPB migrates towards the new daughter bud, whilst the daughter SPB remains in the parental yeast cell (see section “MTOC duplication cycle” for more details). The asymmetric SPB segregation was shown to be regulated by the spindle positioning protein Kar9 as well as the SPB component Nud1, via its role in the mitotic exit network (MEN) (Hotz et al., 2012a). Along the same lines, asymmetric division is also a feature broadly reported in stem cells, in which the cell type of resulting cells –one self-renewed stem cell and one differentiating cell –differs. In *Drosophila* male germ lines, adult stem cells (GSCs) were shown to asymmetrically divide by relying on the inheritance pattern of mother and daughter centrosomes through directional orientation of the mitotic spindle (Yamashita et al., 2003). Using specific labeling techniques, Yamashita and others later observed that the mother centrosome preferentially remains affixed to the GSCs, whilst the daughter centrosome migrates to the differentiating cell (Yamashita et al., 2007). The authors hypothesized that a high number of astral microtubules may be responsible for the anchorage of the mother centrosome to the GSC, thereby keeping them in close proximity during asymmetric cell division. In accordance with this, the predetermined anchoring of the mother centrosome was suggested to act as an orientation mechanism for the mitotic spindle as a way to ensure the success of asymmetric stem cell division and highlights the core role that centrosomes can play in asymmetric stem cell division. Yet, the non-random segregation of mother and daughter centrosomes is not always a prerequisite for spindle alignment and subsequent asymmetric division. After each of the asymmetric divisions undergone by the germline lineage of the nematode *C. elegans*, centrosome rotation occurs as a way to re-align the spindle to the anterior-posterior (AP) axis. This rotation requires that one of the centrosomes, called the leading centrosome and chosen randomly, travels near the anterior border of the cell (Hyman and White, 1987; O’Connell, 2000). This example demonstrates that the non-random segregation of centrosomes during asymmetric division is a common occurrence in some species and does not represent an essential feature of spindle alignment for asymmetric cell division in all biological systems.

Another important function for centrosomes as MTOCs can also be observed in neuronal development. A decisive part of neuronal differentiation lies in axon specification, a process through which one of the neurites matures into a functional axon. This is of high importance for the fate of a neuron, as this process permanently defines its polarization and connectivity. In the current literature, the contribution of centrosomes to this specific stage of neuronal development has met some controversy (as reviewed in Meka et al., 2020). Several reports describe a key role for the centrosome in axonal outgrowth and specification (Rivas and Hatten, 1995; Schaar and McConnell, 2005; Tsai and Gleeson, 2005; Higginbotham and Gleeson, 2007; Kuijpers and Hoogenraad, 2011), whilst other studies seem to contradict such statement and rule out a potential requirement for centrosome function throughout this neuronal process (Esch et al., 1999; Andersen and Bi, 2000; Bradke and Dotti, 2000). In cultured hipoccampal neurons, growing axons were reported to organize microtubule arrays in a centrosome-independent way once axon specification is complete. This observation is supported by the fact that during axonal elongation, centrosome ablation was shown to have no effect on axon extension or regeneration and suggests that centrosomal function may be required only in the earlier stages of neuronal development (Stiess et al., 2010). Recent studies also argue for a role for centrosomes as F-actin organization centers in developing cultured neurons (Meka et al., 2019). Disruption of centrosome function was shown to alter the content of somatic F-actin and decreased peripheral F-actin matter in neuronal growth cones, suggesting a key role for the centrosome in F-actin organization (Meka et al., 2019). During neuronal differentiation, centrosomes as MTOCs can have various other functions. The most classical and well-known function of MTOCs in neuron biology is probably cargo transport across dendrites and axons, a function performed in partnership with motor proteins (Kapitein et al., 2010). In mouse and chick neural tube cells, centrosomes were also shown to influence neuronal delamination, a process by which novel neurons detach from the neuroepithelium throughout differentiation and morphogenesis. For delamination, the centrosome has to be retained in the newborn neuron and nucleates a wheel-like microtubule organization that supports apical abscission. In this process, the centrosome is thus of high importance in mediating microtubule activity and is involved in nervous system growth and expansion (Kasioulis et al., 2017). Another interesting function for centrosomes in neuron biology is in neuronal activity. Using fluorescent microscopy, Hu and others reported that microtubules also have the propensity to invade dendritic protrusions. This observation suggests that MTOCs, through microtubules, may have an implication in the operative exchanges between neurons. An increase in neuronal activity was notably shown to correlate with an increased number of spines occupied by microtubules, as well as with an increased contact time between microtubules and dendritic protrusions (Hu et al., 2008). However, more work is needed to establish the precise function of these microtubules in neuronal plasticity. Taken together, these examples display the various ways in which centrosomes as MTOCs can impact neuronal development and highlight the specialized –and still debated– contribution of this organelle in neuron biology.

#### 1.1.2 Structural Organization of MTOCs

Despite lacking a finite membrane border, the centrosome maintains its unique tri-dimensional shape via centrosome-interacting proteins, 500 of which have been identified to date (Andersen et al., 2003; Gupta et al., 2015; Gheiratmand et al., 2019). Throughout the cell cycle, its size and composition vary, allowing for diverse arrangements in microtubule organization (Devi et al., 2021; Gomes Pereira et al., 2021). Centrosomes contain centrioles, a pair of cylindrical organelles perpendicularly positioned to one another (Figure 1A). Surrounding the centrioles is the pericentriolar material (PCM), a fibrous coiled-coil protein platform (Schatten, 2008) formed by the main microtubule nucleator γ-tubulin, γ-turc, actin (Liu et al., 2020; Wieczorek et al., 2020) and pericentrin proteins (Salisbury, 1995; Levy et al., 1996; Lutz et al., 2001; Martinez-Campos et al., 2004; Wu and Akhmanova, 2017). This platform allows for sustained or transient anchoring of specific signaling proteins, such as the Nuclear Mitotic Apparatus Protein (NuMA), a key effector of the mitotic machinery. Similar to NuMA, centriolin was also reported to connect to the centrosomes during specific phases of mitosis to facilitate cell cycle progression and cytokinesis (Gromley et al., 2003). Importantly, the size of the PCM varies according to levels of γ-tubulin recruited to centrosomes in a way that supports the ongoing cell cycle state. Accordingly, the PCM is a smaller and tighter structure during interphase and becomes much larger during mitosis to support spindle formation through γ-tubulin nucleation (Robbins et al., 1968; Khodjakov and Rieder, 1999).

Aside from this core centrosomal structure comprised of centrioles and their surrounding PCM, other accessory structures including centriolar appendages and satellites positioned around the PCM further decorate centrosomes and provide this essential organelle with extra key features. The mother and daughter centrioles are different in that additional appendages can only be found on the mother centriole. Distal appendages (DAPs), existing at the distal end of mother centrioles across eukaryotic species except for *C. elegans* and *D. melanogaster* (Azimzadeh, 2014), are required for the docking of the centriole to the membrane and for the process of ciliogenesis (Tanos et al., 2013; Ye et al., 2014). Subdistal appendages (sDAPs) are found in close proximity to DAPs and are also involved in cilia formation and microtubule anchoring. In the literature, the relationship between DAPs and sDAPs remains elusive but recent evidence suggests that DAPs are important for sDAPs functionality and positioning (Chong et al., 2020). Apart from these appendages, centrosomes are also surrounded by centriolar satellites, small particles that congregate around the PCM of centrosomes (Kubo et al., 1999). These satellites are mainly composed of proteins involved in the maintenance of centrosomes, neurogenesis and ciliogenesis (reviewed in Prosser and Pelletier, 2020; Odabasi et al., 2020). Centriolar satellites can also play key roles in the transduction of several other biological cues and vary in form and function throughout the cell cycle and across cell types (Kubo and Tsukita, 2003; reviewed in Tollenaere et al., 2015).

Analogous to centrosomes, SPBs of lower eukaryotes act as key microtubule organizing centers but differ dramatically in their mechanism-of-action and structural features (Jaspersen, 2021). Across yeast species, SPBs are functionally conserved but display key architectural differences. Here, we provide a brief description of both budding yeast and fission yeast SPBs as we compare and contrast their organizational features.

**FIGURE 2:**
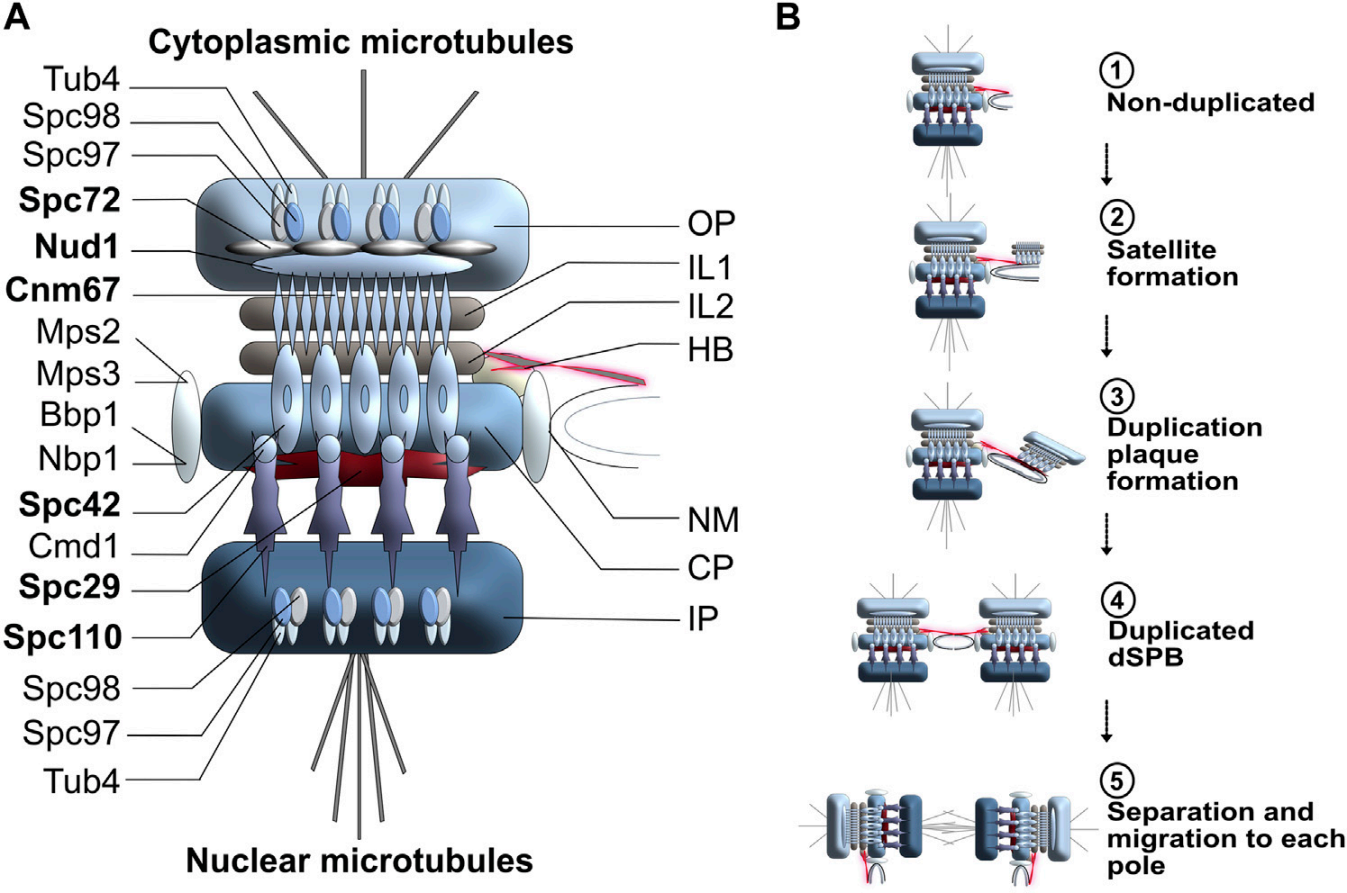
**(A)** Schematic representation of budding yeast SPB organization and duplication cycle. OP, Outer plaque; IL1, Intermediate layer 1; IL2, Intermediate layer 2; HB, Half-bridge; NM, Nuclear membrane; CP, central plaque; IP; Inner plaque. Core SPB components are highlighted in bold. **(B)** SPB duplication cycle in budding yeast. The duplication process of the SPB is conservative and highly dynamic. Step 1: In early G1, the half-bridge is connected to the SPB central plaque and will act as a scaffold for SPB duplication. Step 2: The half-bridge elongates and the core of the daughter SPB (satellite) is generated on the cytoplasmic face of the half-bridge. Step 3: The duplication plaque, resulting from the elongation and growth of the satellite SPB, matures and mimics the cytoplasmic organization of a mature SPB. Step 4: The half-bridge retracts and fuses to the nuclear membrane. The daughter SPB is assembled and is embedded in the nuclear membrane next to the mother SPB. Step 5: The link between mother and daughter SPBs breaks, leading to the separation of the two organelles.

In comparison to the more diffuse centrosomal organization, budding yeast *S. cerevisiae* SPBs are tightly embedded in the nuclear membrane through three highly organized interconnected disk-like structures (see Figure 2A for a detailed representation of budding yeast SPB structure) (Robinow and Marak, 1966; Bullitt et al., 1997; Rüthnick and Schiebel, 2018). The outer plaque is responsible for the nucleation of cytoplasmic microtubules, whereas the inner plaque generates nuclear microtubules. The central plaque anchors the SPB into the nuclear membrane and connects to the half-bridge, an important structure for SPB duplication (Figure 2A). Two tightly packed and organized disks, called intermediate layer 1 (IL1) and intermediate layer 2 (IL2), act as spacers between the outer plaque and the central plaque. In budding yeast, 17 proteins have been identified as SPB structural components (Figure 2A), six of which constitute the core of the spindle pole body: Spc42, Cnm67, Nud1, Spc72, Spc29 and Spc110 (Adams and Kilmartin, 1999; Francis and Davis, 2000; Viswanath et al., 2017). Through reciprocal interactions, these SPB components are integral for creating and maintaining the core SPB structure (Jaspersen and Winey, 2004; Jaspersen, 2021). Most SPB genes are essential for viability and single point mutations in these genes often result in temperature-sensitivity or even lethality.

Fission yeast *S. pombe* SPBs are bipartite structures which, akin to budding yeast SPBs, are implanted in the nuclear membrane. In opposition to budding yeast SPBs, the cytoplasmic domain of fission yeast SPBs represents the bulk of its structure. The architecture of *S. pombe* SPBs also differs from that of budding yeast in that it lacks intermediate spacers and does not contain multiple separate strata except from the outer (cytoplasmic), central and inner (nuclear) layers. Despite these architectural differences, fission yeast SPBs nucleate both cytoplasmic and nuclear microtubules as budding yeast SPBs do and encompass a half bridge required for SPB duplication (Ito and Bettencourt-Dias, 2018). Several components of *S. pombe* SPBs were classified as confirmed or probable homologues of *S. cerevisiae* SPBs, denoting a certain degree of functional and structural conservation in terms of SPB constituents across these species. These include (*S. cerevisiae/S. pombe)*: Tub4/Gtb1; Spc97/Alp4; Spc98/Alp6; Spc110/Pcp1; Spc72/Mto1; Spc42/Ppc89; Cmd1/Cam1; Cnm67/Sid4; Nud1/Cdc11; Cdc31/Cdc31; Sfi1/Sfi1; Mps3/Sad1; Mps2/Kms2. For more details on *S. pombe* SPB and its structural intricacies, we direct readers towards the study of Bestul et al. (2017).

#### 1.1.3 MTOC Duplication Cycle

Centrosome duplication occurs once per cell cycle and is a semi-conservative process (*i.e*., the two centrioles present in each cell duplicate to generate two pairs where one template centriole is older than the newly generated copy; Figure 1B) (Gomes Pereira et al., 2021). Importantly, the process of centriole assembly occurs throughout three full cell cycles. At the onset of replication, procentrioles (new centrioles) separate and by S^1^ phase start their assembly (Figures 1B, step 2). Both procentrioles elongate throughout S^1^ phase, G2^1^ phase and M^1^ phase and grow perpendicularly from their template side (Figures 1B, step 3). At the beginning of M^1^ phase, additional PCM is built around each pair of centrioles as they start to separate (Figures 1B, step 4). To form the mitotic spindle, procentrioles and their developing centrosome separate in early prophase of M^1^. This event, mainly achieved by motor proteins, is supported by push-and-pull forces mediated by the kinesin motor Kif1 and the minus end-directed dynein motor complex. In the literature, the mechanistic intricacies of dynein function in MTOC positioning and separation remained elusive for many years (Holzbaur and Vallee, 1994; Vallee and Sheetz, 1996; Gönczy et al., 1999). Recent studies performed in one-cell *C. elegans* embryos report that different pools of dynein, localized at the cell cortex and on the nuclear surface, can influence centrosome separation. Whilst the pool of dynein located on the nuclear surface moves centrosomes by sliding the centrosome-associated microtubules, the pool of dynein at the cell cortex pulls centrosomes through MT-mediated cortical tugging forces. In this process, dynein was shown to behave as a coupling device that transfers forces produced by polarized actomyosin cortical flows to centrosomes, thereby promoting centrosome separation (De Simone and Gönczy, 2017; Torisawa and Kimura, 2020). Along with this, the plus end-directed kinesin-related motor protein Eg5 creates outward pushing forces by tethering to plus-end antiparallel MTs (Kapitein et al., 2005). Thus, dynein and Eg5 have the ability to create opposite forces that further promotes centrosome separation (Raaijmakers et al., 2012; Agircan et al., 2014). At the end of M^1^ phase, procentrioles are separated and individually assemble their PCM. This occurrence, termed centriole disengagement, signifies that the mother and daughter procentrioles are not in close association anymore. Thus, at this stage of centrosome duplication, disengaged procentrioles can be defined as daughter centrioles. From G1^2^ to S^2^ phase of the following cell cycle, daughter centrioles acquire appendages and further increase in length. Upon entry into S^2^ phase of the second cell cycle, each newly formed daughter centriole begins its own cycle of centrosome duplication once more. During this process, the younger mother centriole persistently accumulates additional PCM from S^2^ phase to G2^2^, until its PCM resembles the older mother centriole PCM prior to M^2^ phase. In G2^2^ phase of the second cell cycle, the younger daughter centriole still develops and acquires distal appendages (DAPs) and subdistal appendages (sDAPs). These appendages will evolve and mature until the G2^3^ phase of the third cell cycle, after which the corresponding round of centriole assembly is complete (Sullenberger et al., 2020).

Centrosome duplication produces two spindle poles that localize perpendicular to the plane of cell division. Achieving this precise orientation is required to support balanced chromosome segregation in mitosis (Kaseda et al., 2012; Silkworth et al., 2012; Nunes et al., 2020). Accordingly, defects in centrosome duplication can have drastic consequences for the cell. If the process of duplication fails and generates extra centrosomes, a resulting scenario may be multipolar mitosis. In multipolar mitosis, chromosomes are segregated to more than two poles during cell division and often leads to gross aneuploidy, chromosome instability (CIN) and clonal evolution (Kwon et al., 2008; Yi et al., 2011; Yang et al., 2012; Telentschak et al., 2015; LoMastro and Holland, 2019). In some cases, clustering mechanisms allow for the formation of a functional bipolar spindle despite the presence of additional centrosomes (Kwon et al., 2008). In other cases, centrosomes may gather at the center of the cell to form a monopolar spindle, a scenario equally threatening to the maintenance of genomic integrity (Chatterjee et al., 2020). Many factors can influence the organization of the mitotic spindle following defective centrosome duplication. Overall, accurate centrosome duplication and partitioning in mitosis is decisive in the maintenance of genome stability and prevention of tumorigenesis.

Like centrosome duplication, SPB duplication is a prerequisite for effective cell division in lower eukaryotes, however, since dynamic exchanges between new and old components occur throughout duplication, SPB duplication cannot be viewed as fully conservative. In budding yeast, the half-bridge elongates in early G1 and remains connected to the central plaque and the IL2 spacer throughout the duplication process (Figures 2B, step 1) (Byers and Goetsch, 1974). Once sufficiently elongated, the daughter SPB is built from satellite material (Figures 2B, step 2), developing into a duplication structure formed by Cnm67, Nud1 and Spc72 through Spc42-directed self-assembly (Winey et al., 1991; Adams and Kilmartin, 1999) (Figures 2B, step 3), after which the half-bridge retracts, allowing for the duplication plaque to embed itself into the membrane. At the end of G1 phase, the parental and daughter SPBs are leveled and connected through a full bridge (Figures 2B, step 4), the disassembly of which allows the parental SPB to preferentially migrate into the daughter bud (Figures 2B, step 5), and form a bipolar metaphase spindle (Roof et al., 1992; Jaspersen, 2021). Following the initial formation of both spindle poles, additional material is added to each SPB in a dynamic manner, hence why SPBs are considered to be dynamic: their growth process should not be viewed as exclusively conservative (Lengefeld and Barral, 2018). Instead, the continuing SPB maturation increases the ability to maintain functional integrity and has been proposed to be a mechanism for SPB repair (Jaspersen and Winey, 2004).

In fission yeast, the process of SPB duplication differs from that observed in budding yeast. The interphase SPB of *S. pombe* localizes in the cytoplasm, in close proximity to the nuclear envelope (NE), and embeds itself in the nuclear membrane only upon mitotic entry. In the literature, the timing of fission yeast SPB duplication throughout the cell cycle has been controversial for many years. Older studies state that SPB duplication occurs in G2/M (Ding et al., 1997), whereas newer studies suggest that the process instead begins in G1/S phase of the cell cycle (Uzawa et al., 2004). When describing SPB duplication and maturation, Uzawa and others separate the maturation process of *S. pombe* SPB into early and late SPB maturation. Early maturation, reported to occur upon S phase completion, represents growth of the lamellae bodies (laminated structure corresponding to the premature SPB) on the half-bridge, nuclear membrane invagination and gathering of material linking the premature SPB to the nuclear membrane. Akin to what is reported in budding yeast, the early event of SPB duplication giving rise to the lamellae bodies in fission yeast relies on the elongation of the half-bridge. The latter, without which SPB duplication could not take place, is required to support the development of the premature SPB. The newly created laminated structure, still undergoing maturation, remains linked to the mother SPB through an ellipsoid bridge (Ding et al., 1997). Late maturation, shown to take place in M phase of the cell cycle, encompasses the separation of mother and daughter SPBs, NE fenestration for SPB insertion and establishment of the mitotic spindle (Uzawa et al., 2004). While individual steps of SPB duplication differ in some respects across yeast species, the process remains broadly conserved overall.

#### 1.1.4 Centrosomes and SPBs: Same, but Different?

**FIGURE 3:**
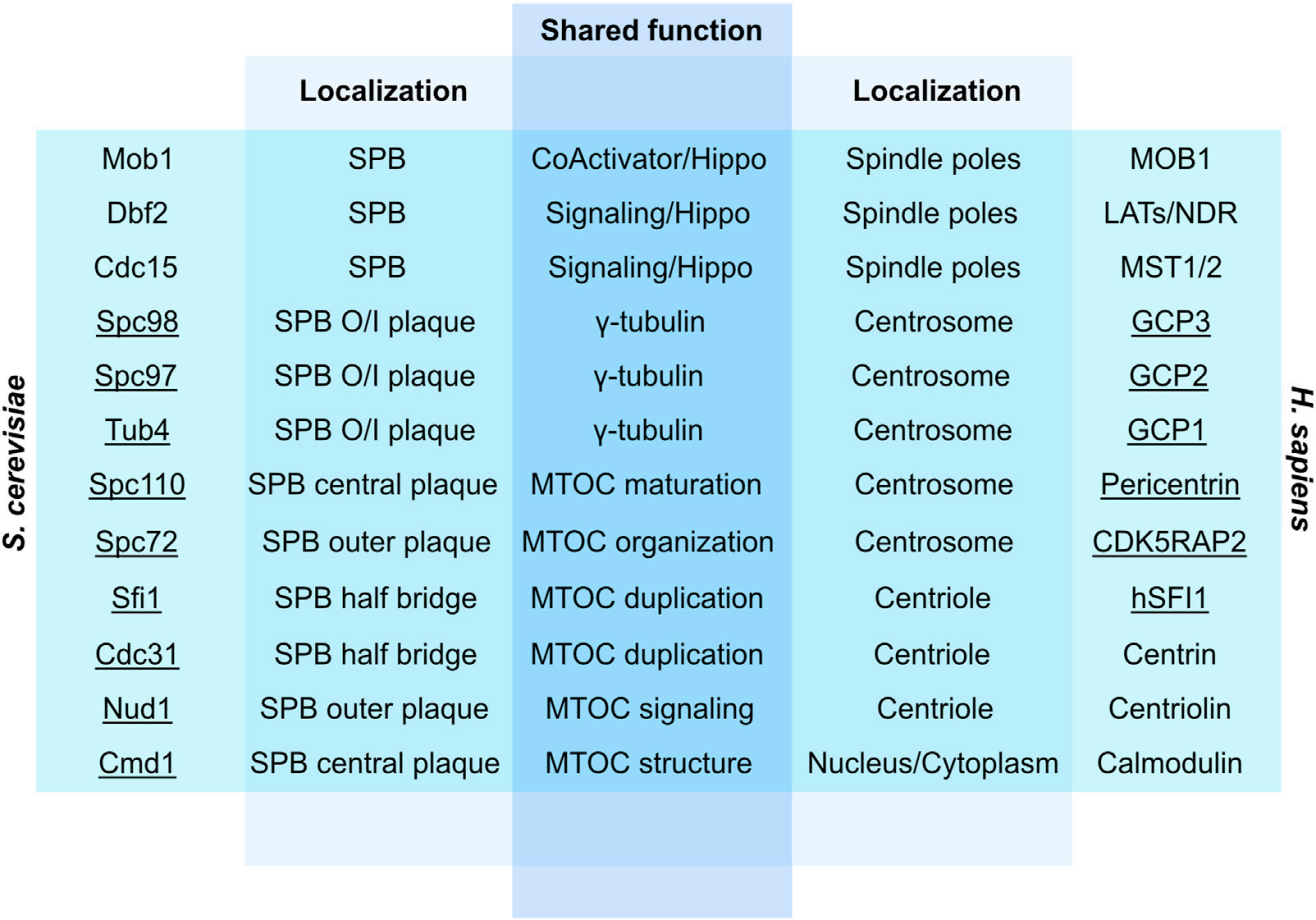
Overview of conserved yeast and human proteins involved in MTOC structure, signaling, duplication and function. Underlined are physical constituents of centrosomes/SPBs. SPB, Spindle pole body; O/I, Outer/Inner; Hippo, Hippo pathway; Pericentrin, Kendrin/CG-NAP (Fraschini, 2019).

Although centrosomes are significantly larger in size than SPBs (Gräf, 2018), they share several characteristics in duplication modes and main MTOC functions (see Figure 3 for centrosome/SPB homologs and orthologs). For example, Kendrin and CG-NAP are human orthologs of yeast Spc110 that localize at the PCM (Flory et al., 2000; Takahashi et al., 2002). Likewise, coiled-coil domains required to establish interactions with analogous binding partners are conserved across yeast Nud1 and human centriolin, both of which are important players in cell cycle progression, mitotic exit and cytokinesis (Gromley et al., 2003; Fraschini, 2019) (Figure 3). However, microtubules nucleated by the centrosome uniquely enables motility, subcellular trafficking, and anchoring of receptors at the surface of the cell (Bettencourt-Dias, 2013), whereas yeast SPBs remain restricted to roles as MTOCs and docking stations for various signaling events.

## 2 Centrosomes as Signal Transduction Organizing Centers

In recent years, an emerging body of evidence support non-canonical roles for centrosomes/SPBs in coordination of signal transduction events (Rincón and Monje-Casas, 2020). Indeed, in response to stimuli and cell cycle cues, kinases with functions unrelated to MTOC activity become transiently enriched at centrosomes/SPBs in a manner that is both necessary and sufficient to promote downstream signaling events. Thus, centrosomes/SPBs can modulate kinase activity in a structural capacity as signal transduction organizing centers (STOCs) (Arquint et al., 2014). This function is analogous to that of supramolecular organizing centers involved in the regulation of innate immunity and programed cell death (Kagan et al., 2014), except that centrosome-mediated events occur at much larger and structurally complex scales. In this section, we will explore the surprising relationship between several kinase families and centrosomes/SPBs and how these organelles act as powerful STOCs.

### 2.1 Centrosomes as STOCs: A Platform to Enable Specialized Functions of Polo-Like Kinases

#### 2.1.1 The Polo-Like Kinase Family

The polo-like kinase (PLK) family, comprised of PLK1-PLK5 in humans, are serine/threonine kinases that regulate fundamental aspects of cell cycle progression (Zitouni et al., 2014; Iliaki et al., 2021). Within this family, polo-like kinase 1 (PLK1) is arguably the most prominent effector of cell cycle events. PLK1 and its functional homologs in budding and fission yeasts (Cdc5 and Plo1, respectively) require phosphorylation by Cdk1/Cdc28/Cdc2 kinases and/or Aurora kinases for full activation *in vivo*. Following this initial activation stage, PLK1 and its yeast counterparts play crucial roles in the regulation of mitotic entry, spindle assembly, chromosome condensation, sister chromatid segregation, cytokinesis, and adaptation to DNA damage (Toczyski et al., 1997; St-Pierre et al., 2009; Ratsima et al., 2011; Zitouni et al., 2014). Importantly, PLK1 function is also essential for centrosome maturation and aberrant PLK1 activity can lead to serious diseases in humans, including cancer (Liu et al., 2017).

All PLKs share a C-terminal polo-box domain (PBD) and a highly conserved multi-domain structure with an N-terminal kinase domain (KD) that harbors a T-loop with an activating phosphorylation site (Rodriguez-Rodriguez et al., 2016; reviewed in; Serrano and D’Amours, 2016). To recognize pre-phosphorylated substrates including CDK1/Cdc28 targets, members of the PLK family use their PBD as a signal amplification module to locate and hyperphosphorylate aforementioned targets. However, the distinctive tripartite architecture of PLK4 PBD differs from the PBDs described across other PLK members and was shown to operate in a phospho-independent manner, making PLK4 PBD an exception on that matter (Slevin et al., 2012). The PBD also behaves as a subcellular targeting domain that allows PLKs to recognize and bind specific structures –such as centrosomes and SPBs– and promote specialized cell cycle functions (Colicino and Hehnly, 2018).

The other members of the PLK family perform distinct but sometimes overlapping functions in cell biology. PLK2, involved in centriole duplication, is dynamically expressed throughout the cell cycle and peaks at the G1/S transition of the cell cycle (Warnke et al., 2004). Given its implication in centriole biology, PLK2 was reported to endogenously localize at the centrosomes throughout the cell cycle. The expression of PLK2 varies widely across tissues and, given its importance in mammary gland development, was shown to be particularly high in mammary tissues (Villegas et al., 2014). On the other hand, PLK3 is more steadily expressed throughout the cell cycle and its function mostly relates to stress response pathways involving p53 during DNA damage and spindle disruption (Donohue et al., 1995; Xie et al., 2001). PLK4, derived from PLK1 (Carvalho-Santos et al., 2010) and sharing with PLK2 a role in centriole duplication, is characterized as a master regulator of MTOC formation and centrosome amplification (Habedanck et al., 2005). The last member of the PLK family, PLK5, has a slightly different structure than other members of its group in that it completely lacks a kinase domain in humans. Opposite to its other orthologs, the expression of PLK5 was shown to be very low throughout cell division and high in quiescent cells. PLK5 expression is highest in brain tissues and plays a core function in the nervous system, including neuron differentiation (de Cárcer et al., 2011a). For more information regarding the PLK family, its family members and its evolution across species, we direct readers towards reviews covering these topics (Archambault and Glover, 2009; de Cárcer et al., 2011b).

**FIGURE 4:**
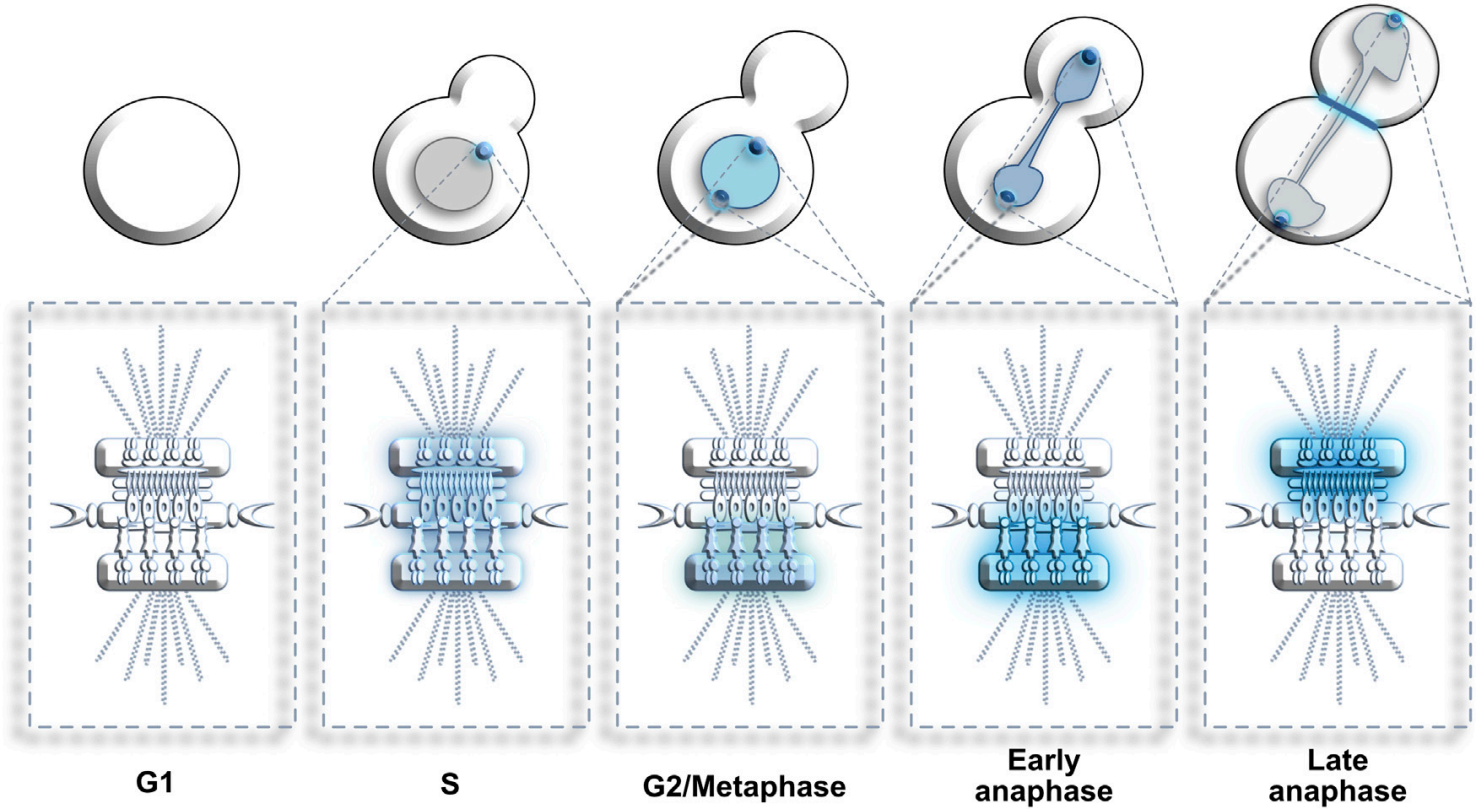
Dynamic localization of Cdc5/Polo kinase at SPBs. G1: Cdc5 is absent from cells. S: Cdc5 enriches at the non-duplicated SPB. G2 to metaphase: Cdc5 decorates the nucleus and the nuclear surface of both SPBs. Early anaphase: Cdc5 concentration at the nuclear surface of both SPBs increases. Late anaphase: Cdc5 relocates from the inner to the outer surface of both SPBs (and bud neck) where it stimulates mitotic exit. Blue color represents enrichment of Cdc5. Color intensity represents Cdc5 concentration levels.

MTOCs are crucial scaffolding structures used by PLKs to reach specific substrates and promote cell division (Figure 4). In *S. cerevisiae*, Cdc5 decorates the nuclear surface of duplicating SPBs from late S phase to early anaphase and is also located in the nucleus. In late anaphase, Cdc5 enriches specifically on the cytoplasmic side of the parental SPB segregated to the daughter bud as well as on the bud neck (Botchkarev and Haber, 2017). Once the cell cycle is completed, Cdc5 is degraded by the anaphase-promoting complex (APC) throughout the G1 phase of the next cell cycle (Visintin et al., 2008).

In fission yeast, the polo-related kinase Plo1 shows equally important roles in cell cycle progression and displays high levels of functional overlap with budding yeast Cdc5 and human Plk1 (Lee et al., 2005). Amongst its key roles, Plo1 is required for mitotic entry, formation of the mitotic spindle, establishment of the actin ring prior to cytokinesis as well as septation activation preceding mitotic completion (Ohkura et al., 1995). Similar to Cdc5 and Plk1, Plo1 requires the SPBs as a docking platform and transiently enriches on the structure in a spatiotemporal manner throughout the cell cycle. Similar to the enrichment of Cdc5 at the SPBs, which is low in S-phase but high in G2/M (Simpson-Lavy and Brandeis, 2011), the enrichment of Plo1 on the SPBs is high during mitosis but absent in interphase. Additionally, Plo1 activity at the SPBs is highly reliant on the activity of the kinase Cdc2 (Mulvihill et al., 1999). Upon Cdc2 activation, Plo1 enriches at the SPBs and remains until spindle breakdown whilst keeping steady expression levels throughout the cell cycle (Lee et al., 2005).

The enrichment of Plo1 at the SPBs plays a pivotal role in the commitment to cell division and mitotic progression. The process of mitotic commitment is tightly regulated by M-phase promoting factor (MPF) (Ohi and Gould, 1999), composed of the regulatory subunit cyclin B and the catalytic subunit Cdc2. Following its recruitment to the SPBs in G2 phase (Alfa et al., 1989; Decottignies et al., 2001; Grallert et al., 2013), MPF activity can promote mitotic entry at any point during the cell cycle. Consequently, its activity must remain strictly restrained to the instant where cell division is timely and suitable. The kinase Wee1, via the phosphorylation of Cdc2, is responsible for such inhibitory effect on MPF activity (Russell and Nurse, 1987). Once all conditions for mitotic progression have been fulfilled, the phosphatase Cdc25 removes the inhibitory phosphorylation on Cdc2 and thus promotes cell division. Once MPF is activated, the complex creates a positive feedback loop that further promotes mitotic commitment through increased Cdc25 activity and Wee1 inhibition. Downstream of this feedback loop instigated by MPF, Plo1 interacts with the SPB component Cut12 in a way that supports entry into mitosis. The NIMA-related kinase Fin1, along with Plo1, was also reported to contribute to this positive feedback loop (Grallert and Hagan, 2002).

Apart from its involvement in mitotic entry, Plo1 is equally important throughout cell division. Plo1 shows two mechanistically distinct activity peaks during mitotic progression: First during prophase, where the formation of the actin ring occurs; second in late mitosis, corresponding to septum formation (Tanaka et al., 2001). Indeed, Plo1 was reported to localize to the medial ring structures as soon as they arise, a subcellular zone that correlates with its key function in the setting of partition sites (Bähler et al., 1998). Akin to Cdc5 in budding yeast, fission yeast Plo1 relies on the APC for its disassociation from the SPBs upon mitotic completion (Mulvihill et al., 1999). Overall, the enrichment of Plo1 at the SPBs is reflective of its implication in the spatial organization of mitotic processes and represents an essential step in the regulation of mitotic entry and cell cycle progression (Lee et al., 2005; Grallert et al., 2013).

In human cells, the Aurora-A kinase, in complex with its co-factor Bora, phosphorylates Plk1 on a conserved residue located in the T-loop of its kinase domain (T210). This G2 phase phosphorylation event uniquely occurs at the centrosomes (Bruinsma et al., 2015). Throughout the cell cycle, Plk1 localization and activity varies greatly. In late G2/early prophase, Plk1 preferentially enriches at the centrosomes to promote mitotic entry and then becomes enriched at the kinetochores to support microtubule-kinetochore connections in prometaphase, with lower Plk1 levels remaining at the centrosomes to instruct spindle assembly.

#### 2.1.2 PLK1 in the DNA Damage Response

The dynamic localization of Cdc5/Plk1 at MTOCs has major implications for signal transduction events during the cellular response to DNA damage. Upon DNA damage, cells initiate a checkpoint response that allows time for DNA repair by preventing the G2/M transition (Sandell and Zakian, 1993; Rhind and Russell, 1998; Cagney et al., 2006; Chao et al., 2017). After successful DNA damage repair, cells resume cycling through a process termed checkpoint recovery (Vaze et al., 2002). However, not all DNA lesions can be safely repaired, and the extent of damage suffered determines the fate of the damaged cells. When DNA damage is too extensive, apoptotic signals lead to programmed cell death thereby preventing the transfer of deleterious genomic errors to daughter cells. When DNA damage is less extensive, cells can resume their cell cycle through checkpoint adaptation (or bypass) despite the presence of “permanent” DNA damage (Sandell and Zakian, 1993; Toczyski et al., 1997; Lee et al., 1998; Vidanes et al., 2010; Ratsima et al., 2011). Consequently, the process of checkpoint adaptation postpones the repair of DNA lesions to subsequent phases of the cell cycle.

The exact signaling pathway responsible for the adaptation response to persistent DNA damage is still not fully understood. In both human and yeast cells, PLK activity is required for adaptation, and Cdc5 enrichment at the SPBs is both necessary and sufficient to promote adaptation to persistent DNA damage in budding yeast cells (Ratsima et al., 2016). These observations suggest that SPBs function as docking platforms for Cdc5 to execute the adaptation response. How this is achieved is unclear, however a possible link connecting PLK, BRCA1 and centrosomes was recently proposed in human cancers (Yoshino et al., 2021). In some cases, aberrant expression of the tumor suppressor gene *BRCA1* in mammary tissues can dysregulate centrosome duplication and generate a higher centriole number *in vivo*. This reported process requires the tethering of BRCA1 to centrosomes via RACK1. This protein also acts as a scaffolding factor that promotes Aurora A and PLK1 interaction in S phase. Previous literature linked RACK1 overexpression to centriole overduplication and involved BRCA1 as a component in this process (Yoshino et al., 2019; Yoshino et al., 2020). This centriole overduplication event was shown to stem from higher levels of phosphorylated PLK1, resulting in kinase hyperactivity at centrosomes. The reported centrosome aberration phenotype in response to PLK1 hyperactivity is intriguingly reminiscent of the supernumerary SPB and polyploidy/multinucleation phenotypes observed in adaptation-defective *cdc5-16* mutants (Ratsima et al., 2011; Ratsima et al., 2016) and in cells overexpressing *CDC5* (Song et al., 2000; Bartholomew et al., 2001). However, more research is needed to assess whether there are cross-species phenotypic similarities between these two cellular processes and how this might be related to the adaptation response to unrepairable DNA damage. Despite the impact of BRCA1 aberrations reported above in centrosome amplification (Yoshino et al., 2019; Yoshino et al., 2020), other studies demonstrated that mutations in BRCA1 can induce a variety of phenotypes that do not always result in amplified centrosomes. To further evaluate the influence of BRCA1 in centrosome biology *in vivo,* Kais and others explored the effect of a subset of mutations in the *BRCA1* locus on centrosome behavior. Remarkably, these mutations induced a range of phenotypes affecting two separate branches of centrosome biology, namely centriole pairing and centrosome number. This result suggests that BRCA1 regulates these two branches of centrosome duplication separately, and nicely underlines the separation-of-function aspect of certain BRCA1 mutations (Kais et al., 2012). Thus, some mutations in BRCA1 can affect functions unrelated to centrosome number and do not always correlate with centrosome amplification in transformed cells.

The process of DNA damage-induced centrosome amplification (DDICA) (Zou et al., 2014) represents another intriguing link connecting DNA damage responses, PLKs and MTOCs. After treatment with the DNA crosslinker mitomycin C, higher levels of BRCA1 and PLK1 were detected at centrosomes alongside increased centrosome amplification. How DDICA might enhance genomic stability and/or survival remains unclear to this day. On one hand, DDICA may promote the elimination of cells bearing extensive amounts of DNA damage through mitotic catastrophe, whilst contributing to DNA damage repair via local increase of DNA repair factors at the centrosomes (Yoshino et al., 2021). The rationale behind this is that an increased amount of DNA repair factors at the centrosomes stemming from DDICA could constitute an extra source of DNA repair proteins available for relocation from the centrosomes to nuclear sites of DNA damage, consequently supporting nuclear DNA repair as well as DDICA processing. This theory, however, remains to be proven and is a work in progress in the current literature. On the other hand, this process was suggested to be beneficial for cancer cells seeking a proliferative advantage in specific growth environments, as centrosome amplification in p53-deficient cancer cells can encourage chromosome mis-segregation (Yoshino et al., 2021), a key promoter of genomic heterogeneity. The mitotic catastrophe phenotype resulting from DDICA, observed primarily in breast cancer cells, is intriguingly evocative of the phenotype reported in budding yeast with the adaptation-defective *cdc5-16* allele. In response to DNA damage, this mutant fails to enrich at the SPB and gradually fragments its SPB, akin to DDICA (Ratsima et al., 2011). It would be informative for future research to explore the mechanistic similarities between Cdc5-related SPB fragmentation in yeast and PLK1/BRCA1-related DDICA in breast cancer cells.

In damaged cells, the generation of extra centrosomes can also be an outcome of circumstances unrelated to PLK1 or BRCA1 expression. Dodson and others notably reported that centrosome amplification can ensue an extended G2 phase caused by DNA damage checkpoint activation, in which DNA replication is paused but centrosome duplication remains. Interestingly, the small portion of cells able to override this G2/M cell cycle arrest were shown to contain a normal number of centrosomes (Dodson et al., 2004). Other potential causes of centrosome amplification also include cytokinesis failures, as well as cell-cell fusion (reviewed in Godinho and Pellman, 2014). Overall, the relationship between centrosome amplification and DNA damage is an ongoing work in progress in the field of centrosome biology and its intricacies are yet to be fully uncovered.

### 2.2 Centrosomes as STOCs: PIDDosome Signaling Axis and the Centrosome Surveillance Pathway

Centrosome biogenesis is a process finely coordinated with other cell cycle cues to minimize errors during centriole duplication. In some cases, this control system fails despite its global efficacy and consequently leads to aberrations in centrosome biogenesis. In the literature, centrosome aberrations sometimes are described as a common outcome of neoplastic transformation (LoMastro and Holland, 2019). However, research shows that these aberrations can in fact be at the core of neoplasia, acting as an instigator of cell transformation (Lingle et al., 2002; Pihan et al., 2003; Segat et al., 2010; Lopes et al., 2018; Burigotto et al., 2021). In recent years, a link between centrosomes and the tumor suppressor p53 was unraveled and pointed to a control system for centrosome biogenesis. This control system, termed the PIDDosome signaling axis, acts as a mitotic clock that can detect and react to centrosome aberrations and DNA damage during cell proliferation to monitor and minimize genomic instability (Tinel and Tschopp, 2004; Ando et al., 2012; Ando et al., 2017; Fava et al., 2017; Sladky et al., 2017; Tsabar et al., 2020). The PIDDosome signaling axis is composed of the “cell-death effector caspase-2” (CASP2), the “p53-induced death domain-containing protein 1” (PIDD1) as well as the “CASP2 and RIPK1 domain containing Adaptor with Death Domain” (CRADD). In response to stress signals such as extra centrosomes or genotoxic insults, the local concentration of centrosomal PIDD1 increases and specifically enriches at the mother centriole via ANKRD26, a distal appendage protein (Burigotto et al., 2021). Processing of PIDD1 at the centrosome via auto-cleavage leads to its release in the cytoplasm, where the auto-catalytic and proximity-driven activation of CASP2 occurs (Tinel and Tschopp, 2004). Resulting CASP2 activity stimulates the cleavage of the E3 ubiquitin-ligase MDM2, a negative regulator of p53 stability, ultimately leading to the activation of the tumor suppressor p53 and upregulation of p21, a cell cycle inhibitor (Oliver et al., 2011). To limit cell proliferation, this sequence of events leads to a cell cycle arrest or cell death and thereby supports the maintenance of genomic stability (Evans et al., 2021). The increased local recruitment and resulting enrichment of centrosomal PIDD1 at the distal appendages of the mother centriole is suggested to stem from a cellular surveillance mechanism, in which an abnormally high number of mature centrioles can stimulate the activation of the PIDDosome signaling axis (Fava et al., 2017).

Similarly to the PIDDosome signaling axis in response to centrosome amplification, another pathway termed the centrosome surveillance pathway monitors and reacts to centrosome loss or prolonged mitosis (Lambrus et al., 2016; Meitinger et al., 2016; Lambrus and Holland, 2017). In response to disturbed mitosis, the scaffolding protein 53BP1 acts as a platform to recruit the protein deubiquitinase USP28 as well as p53. The resulting proximity between USP28 and p53 leads to the deubiquitination and subsequent change in p53 activity and p21 upregulation, leading to a proliferation arrest in G1 phase of the cell cycle (Fong et al., 2016; Lambrus et al., 2016; Meitinger et al., 2016). The mechanistic intricacies responsible for the activation of the centrosome surveillance pathway are not fully understood. However, variations in PLK4 expression and activity appear to be linked to centrosome loss and subsequent activation of the centrosome surveillance pathway (Wong et al., 2015). Despite both 53BP1 and USP28 proteins being known binding partners involved in DNA damage response pathways (Zhang et al., 2006; Knobel et al., 2014; Panier and Boulton, 2014; Zimmermann and de Lange, 2014), evidence shows that the activity of the centrosome surveillance signaling pathway is independent from their canonical functions in DNA damage and uncovers a new separate line of defense against the loss of genomic integrity (Lambrus et al., 2016).

### 2.3 Centrosomes as STOCs: Regulation of Mitotic Entry by cAMP-Dependent Protein Kinase A

**FIGURE 5:**
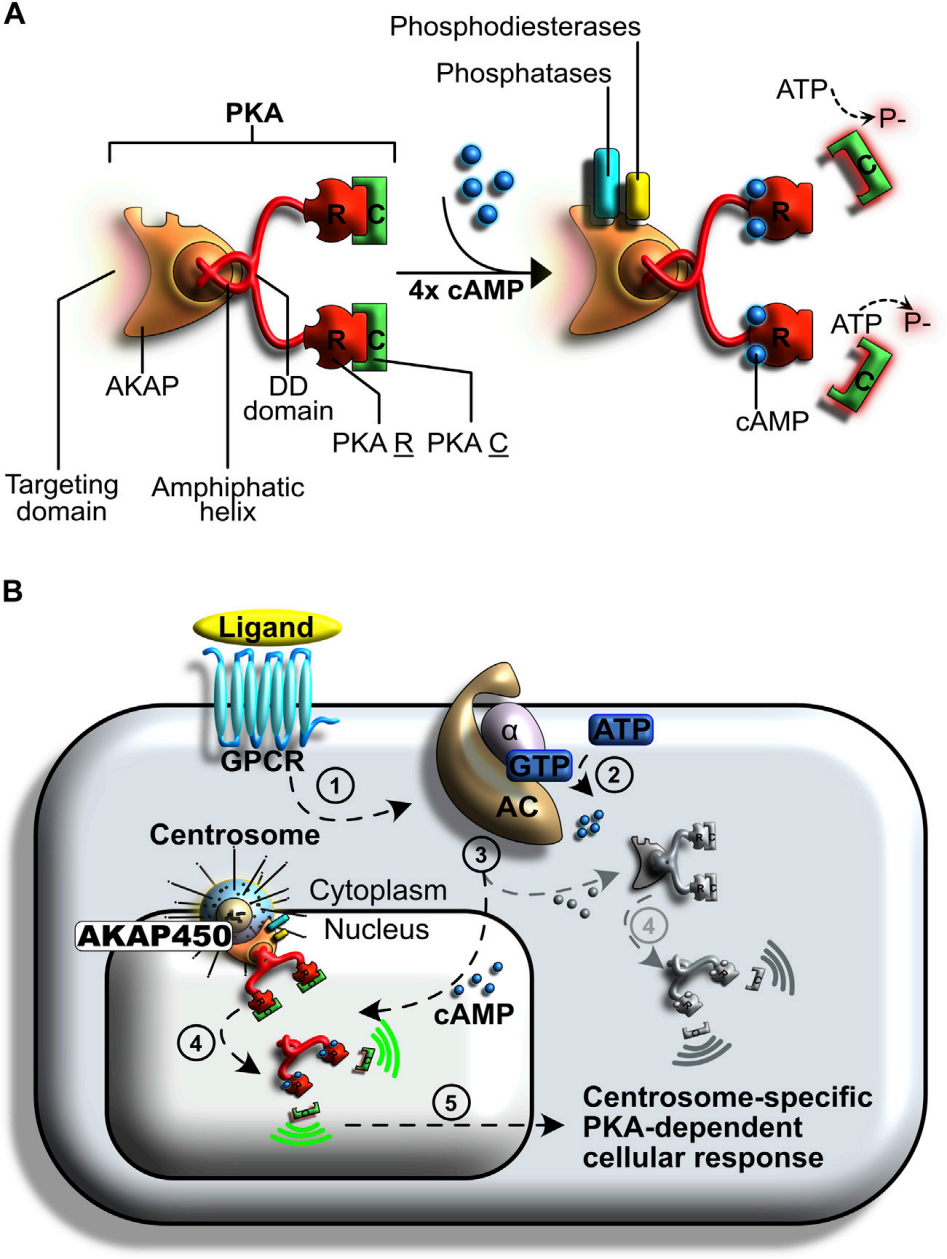
Centrosome-specific regulation of protein kinase A (PKA) signaling. **(A)** PKA is a tetrameric holoenzyme composed of two regulatory subunits and two catalytic subunits. Its activity relies on cyclic AMP (cAMP) cellular levels and is involved in many regulatory processes. **(B)** Regulation of PKA following G protein-coupled receptor (GPCR) activation. A ligand binds to the GPCR (step 1), initiating the signal transduction cascade. This signal induces a GDP to GTP exchange on a heterotrimeric G complex (step 2). The Gα subunit is released and binds to adenylyl cyclase (AC), an event that induces the formation of cyclic adenosine monophosphate (cAMP) from ATP. A subpopulation of PKA anchors at the centrosomes (step 3). The resulting AKAP450 complex increases PKA affinity for cAMP. Centrosomal PKA is selectively activated by cAMP, whilst cytosolic PKA (shown in grey) remains mostly inactive (step 4). A specialized cellular response is induced by the catalytic activation of PKA at centrosomes (step 5).

Recent work has revealed that PKA activation is regulated differentially in distinct subcellular compartments, and that localized activation sites –known as signaling islands– are key in determining the profile of substrates modified by this kinase (reviewed in Omar and Scott, 2020). PKA localization and its activation kinetics at centrosomes are controlled by kinase-anchoring proteins (AKAPs). Specifically, AKAP450-controlled autophosphorylation of the PKA regulatory subunit lowers the cAMP threshold required for PKA activation at centrosomes (Figures 5A,B) (Taylor et al., 1990; Di Benedetto et al., 2008; Taylor et al., 2008; Terrin et al., 2012). In parallel, cAMP-specific phosphodiesterase (PDE4D3) maintains a low cAMP concentration in the vicinity of this organelle. Combined, these two mechanisms allow for a restricted centrosomal PKA pool to maintain activity when cytosolic PKA is mostly inactive, and thereby promote cell cycle progression without inadvertently inducing gene transcription, signal transduction, or other undesired events.

At the onset of cell division, mitogenic signals trigger an increase in cAMP levels in the entire cell, including the centrosome (Vandame et al., 2014). The increase in centrosomal cAMP is believed to be partly induced by MAPK-mediated inhibition of PDE4D3, which allows the concentration of cAMP to increase (Terrin et al., 2012). However, an exogenous increase in global cellular cAMP levels is not sufficient to induce PKA-mediated cell cycle progression to promote mitosis. Instead, an increase in centrosomal cAMP levels is required; when AKAP450 is artificially relocated away from centrosomes, lack of PKA impairs mitosis and leads to a block in G1 (Gillingham and Munro, 2000; Keryer et al., 2003). Conversely, an artificial increase of centrosomal cAMP levels induces a buildup of prophase cells (Terrin et al., 2012; Vandame et al., 2014).

Together, these studies unraveled that selective activation of centrosomal PKA is pivotal for inducing the cAMP-dependent pathway during mitosis. In this setting, centrosomes act as supramolecular docking platforms in which conditions for PKA activation differ significantly from those that prevail elsewhere in the cell.

### 2.4 Centrosomes as STOCs: Regulation of Cell Proliferation Decisions by NIMA-Related Kinases

NIMA-related protein kinases are serine/threonine kinases involved in multiple MTOC-related processes. In metazoans, these processes include centrosome separation, during which centrosomes migrate to opposite poles of the cell, spindle assembly, and MTOC-independent regulation of mitotic checkpoints (Nigg, 2001; O’Connell et al., 2003; Moniz et al., 2011; Fry et al., 2012). In humans, seven NIMA-related kinases (Neks) have been identified, whereas lower eukaryotes typically encode a single family member.

The Nek2 isoform in humans is enriched at the centrosomes. Although Nek2 associates with centrosomes in all stages of mitosis, independently of microtubules, its activity is highest in S and G2 phases (Fry et al., 2012). Nek2 is required for centrosome integrity, as evidenced by dramatic phenotypes caused by loss or gain of function mutations. Loss of function mutations were reported to impair centrosome disjunction, a process through which the proteinaceous linker keeping the mother and daughter centrioles in close proximity normally disappears (Hinchcliffe and Sluder, 2001; Fu et al., 2015), and to elicit the formation of monopolar mitotic spindles (Faragher and Fry, 2003; O’Regan et al., 2007). On the other hand, gain of function mutations were reported to induce premature centrosome splitting where a single centrosome would separate into two distinct foci, gradual centrosome loss, and dispersal of centrosomal material (Fry et al., 1998; Fang and Zhang, 2016). Beyond its MTOC-dependent role, Nek2 promotes chromatin condensation in mouse meiotic spermatocytes (Di Agostino et al., 2004; Fry et al., 2012) and cytokinesis in *Drosophila* (Prigent et al., 2005). In fission yeast, the unique Nek2 homolog Fin1 likewise contributes to key cellular processes ranging from mitotic commitment (see section *“Centrosomes as STOCs: Polo-like kinases–The Polo-like kinase (PLK) family”* for an overview of mitotic commitment in *S. pombe*) to spindle function, maintenance of nuclear envelope dynamics and regulation of the septum initiation network (SIN) (Krien et al., 2002; Grallert et al., 2004). Certain phenotypes observed across species upon gain or loss of function mutations in NIMA-related kinases share common themes. Fin1 overproduction in *S. pombe* was notably reported to create spindle formation defects, reminiscent of the centrosome splitting phenotype associated with Nek2 gain of function mutations in humans (Fry et al., 1998; Krien et al., 2002). Despite the lower amount of functional overlap observed in this class of protein kinases in comparison to others (such as PLKs) across species, NIMA-related kinases still share several functional features from yeast to humans and represent an important class of proteins with vital functions in cell biology.

#### 2.4.1 Nek2-Mediated Signaling in the Wnt/Wingless Pathway

Nek2 is known to phosphorylate *ß*-catenin, a multifunctional Wnt-pathway effector implicated in a wide array of biological contexts including centrosome-related cellular processes (Kaplan et al., 2004; Bahmanyar et al., 2008; Vora et al., 2020). Throughout mitosis, Nek2-mediated *ß*-catenin phosphorylation prevents its degradation, a mechanism required to maintain high levels of centrosomal *ß*-catenin (Mbom et al., 2014) and associated with accurate centrosome disjunction. Nek2 kinase activity at the start of mitosis relies on Plk1 (Mardin et al., 2011), however, *ß*-catenin enrichment at the centrosomes is independent of its phosphorylation state (Mbom et al., 2014). Outside of the centrosome, the Nek2b isoform forms a complex with T-cell factor (TCF4) to drive *ß*-catenin-dependent cell proliferation, a mechanism associated with tumor cell invasion and metastasis (Shin et al., 2017; Zhang et al., 2017; Shen et al., 2019).

Nek2 also phosphorylates dishevelled (DVL), a scaffold protein involved in both the canonical and non-canonical branches of the Wnt pathway. In higher organisms, three genes encode DVL isoforms –*DVL1*, *DVL2* and *DVL3*. These isoforms, broadly expressed in mammalian cells, were reported to have partly overlapping functions with high levels of redundancy (Lee et al., 2008). The phosphorylation event mediated by Nek2 on DVL isoforms is essential to promote interactions between DVL and several centrosomal linker proteins, liberating these from the centrosomes and ultimately promoting centrosomal separation. Indeed, lack of DVL impedes the dissolution of centrosomal linkers, resulting in an absence of centrosomal separation (Cervenka et al., 2016). Nek2 can also positively modulate the pool of DVL available at the centrosomes to upregulate the canonical Wnt/β-catenin pathway (Cervenka et al., 2016).

Apart from its implication in centrosome separation, the Wnt signaling pathway was also reported to play a role in cell motility. In response to exosome-transported signaling molecules named planar cell polarity (PCP) proteins, the Wnt pathway stimulates breast cancer cell (BCC) motility at the cell cortex. For this event to occur, the association of a centrosomal module is required. Specifically, DVL2 isoform was shown to mediate the assembly of this module, composed of the human centrosomal protein CEP192 and PLK4/AURKB, to promote protrusive activity in BCCs. This centrosomal module coordinates the exchange of formin DAAM1 for formin DAAM2 at the cell cortex, resulting in increased cell migration (Luo et al., 2019). This sequence of events may partly explain why aberrant expression of PLK4, AURKB and DAAM2 in breast cancer was shown to correlate with poor prognosis and increased cancer aggressiveness (http://www.cbioportal.org). Interestingly, the function of this centrosomal module was reported to be independent of centrosomes or microtubules and elegantly highlights how contextual Wnt signalling in cancer cells has the power to initiate processes such as cell migration as a means to augment metastatic potential.

In the developing *Drosophila* eye, the relationship between Nek2 and Wnt/Wingless is more direct. In a setting where the anaphase-promoting complex (APC) is inactivated, Nek2 accumulation causes hyperactivation of Wnt signaling and blocks retinal differentiation. Conversely, when Nek2 is degraded by APC, local Wnt signaling is suppressed and retinal differentiation proceeds (Martins et al., 2017). Taken together, these studies highlight how Nek2 operates in partnership with the Wnt pathway throughout the entire cell, including at the centrosomes.

### 2.5 Centrosomes as STOCs: Regulation of Mitotic Exit and/or Cytokinesis by MEN and SIN Kinases

The mitotic exit network (MEN) is a GTPase signaling cascade that regulates cell cycle progression in budding yeast with similarities to the Hippo signaling pathway in metazoans. MEN drives the onset of mitotic exit in late anaphase and cytokinesis primarily by inhibiting the activity of Cdk1 and reversing phosphorylation sites on Cdk1 substrates. SPBs provide spatio-temporal cues for MEN, and importantly, functions as docking platforms to initiate and amplify signaling events.

Up until anaphase, the GTPase Tem1, the main MEN initiator, is present at SPBs but is kept inactive until Cdc14 phosphatase is released from the nucleolus to create a positive feedback loop that drives the mitotic exit process (as reviewed in Manzano-López and Monje-Casas, 2020). Two spindle position checkpoint (SPOC) components, GTPase-activating proteins (GAP) Bfa1/Bub2, inhibit Tem1. In anaphase, spindle elongation allows the older SPB to progressively migrate from the mother cell into the daughter bud, at which point the Cdc5 kinase, enriched at the SPBs, phosphorylates Bfa1/Bub2 to disinhibit Tem1. Concomitantly with this, migration of the older SPB into the bud places the Lte1 guanine-exchange factor (GEF), located in the bud cortex, where it can access and convert Tem1 to its active GTP-bound form. Subsequently, the Cdc15 kinase and its downstream effector –the Dbf2-Mob1 complex– are recruited to SPBs and activated, allowing transmission of the MEN signal to the nucleolus, where it can activate Cdc14 (Renicke et al., 2017; Campbell et al., 2019).

The release of Cdc14 and its gradual accumulation outside of the nucleolus generates a robust feedback loop that promotes mitotic exit (Barberis et al., 2005; Maekawa et al., 2007). Cdc14 enriches at the SPBs via its interaction with the outer plaque component Spc72, and throughout anaphase, gradually increases on the parental/older SPB as it migrates through the daughter bud (Yoshida et al., 2002). In late telophase, once the daughter SPB is fully generated, Cdc14 accumulates on both SPBs. By acting as a docking platform for Cdc14, SPBs may act as a functionally distinct reservoir of active Cdc14 responsible for promoting effective mitotic exit (Yoshida et al., 2002).

The wealth of knowledge on the MEN and its role in mitotic exit sometimes overshadows its equally important roles in cytokinesis. In budding yeast, establishment of an actomyosin ring and septum formation between the mother and daughter bud at the beginning of anaphase are necessary processes for completing cell division and separate the two newly formed cells. Given the temporal pairing of late mitotic events and cytokinesis, many MEN components are also required for the completion of the cytokinetic process. Amongst them, SPB-bound Tem1 and the Bfa1/Bub2 complex were shown to be crucial for successful cytokinesis (Whalen et al., 2018) and the activity of the SPB-enriched Cdc5 kinase required to complete cytokinesis. In late anaphase, Bfa1 maintains Cdc5 mainly on the cytoplasmic side of the daughter SPB (Park et al., 2003). At the onset of cytokinesis, Cdc5 gradually enriches at the bud neck and promotes cell division through its kinase activity towards a specific subset of substrates. The preferential enrichment of Cdc5 at the outer side of the daughter SPB seemingly facilitates the late mitosis/cytokinesis transition by allowing for the rapid migration of Cdc5 at the bud neck (Botchkarev et al., 2017). Thus, the role played by SPBs as platforms that coordinate MEN signaling has implications beyond the area of mitotic exit, such as the regulation of key events required for the completion of cytokinesis.

The septation initiation network (SIN) in fission yeast, a GTPase signaling cascade akin to the budding yeast MEN, regulates several mitotic processes occurring in the last steps of cell division. These processes include actomyosin ring constriction (CAR), septation and cytokinesis (Feoktistova et al., 2012; Alcaide-Gavilán et al., 2014; Edreira et al., 2020). The first event leading to SIN initiation requires the activation of the Ras-like GTPase Spg1 (septum-promoting GTPase) (Schmidt et al., 1997). In metaphase, both SPBs contain uniform amounts of Spg1. The latter, ensuing its activation, recruits its effector protein kinase Cdc7 at the SPBs. Upon anaphase entry, both Spg1 and Cdc7 become inactivated on the parental SPB whilst Cdc7 concentration increases on the daughter SPB (Sohrmann et al., 1998). The resulting asymmetrical enrichment of Cdc7 on the newer SPB further induces the recruitment of Sid1 and Sid2 protein kinases on the daughter SPB, stimulating SIN activity and contributing to the transduction of septation signals from the SPB to the division site (Guertin et al., 2000). Furthermore, Sid2 was reported to exert a positive effect on SIN activity feedback loop, thus maximizing the signaling cascade to promote septation (Feoktistova et al., 2012). The polo-like kinase Plo1, involved in several steps of mitotic progression, was also reported to positively impact SIN activity and was hypothesized to operate upstream of the aforementioned signaling cascade (Ohkura et al., 1995; Tanaka et al., 2001). Loss-of-function mutations encompassing certain *SIN* genes were reported to induce the formation of elongated multinucleated cells, resulting from the absence of cell division following several cycles of nuclear division (Nasmyth and Nurse, 1981; Balasubramanian et al., 1998). Conversely, gain-of-function mutations were linked to the establishment of numerous actomyosin contractile rings and septa in cells without divided nuclei, a consequence of mutated inhibitors of SIN (Feoktistova et al., 2012).

The function of the MEN in mitotic exit represents a late evolutionary trait. Since the regulation of mitotic exit was coupled to the mitotic exit (ME)-signaling pathway only during the development of the Saccharomycetaceae family, other yeast species such as *C. albicans* or *S. pombe* thus lack this function of the MEN (Maekawa et al., 2022). Moreover, it is worth noting that the MEN was suggested to function earlier in the cell cycle, such as in metaphase, in other processes unrelated to mitotic exit and cytokinesis. SPBs were notably reported to exploit the MEN as a way to drive age-dependent segregation. The spindle positioning protein Kar9 was shown to impact SPB segregation through preferential asymmetric enrichment to the older SPB in metaphase, a process requiring sustained Kar9 phosphorylation by the MEN kinases Dbf2 and Dbf20 (reviewed in Hotz and Barral, 2014). The SPB component Nud1 was also reported to further support the asymmetric enrichment of Kar9 on the old SPB and demonstrates that the MEN can impact cell cycle progression as early as in metaphase, through the establishment of asymmetric SPB inheritance (Hotz et al., 2012a; Hotz et al., 2012b). Importantly, the contribution of the MEN to early mitotic events was shown to be conserved across several eukaryotic species, including *S. pombe*, and suggests that this specific feature of the MEN is a commonly shared evolutive trait (Hachet and Simanis, 2008; Chiba et al., 2009; Chiyoda et al., 2012; Grallert et al., 2012). Despite the fact that cell cycle progression is a collective function of Hippo-related kinases across many eukaryotic species, exceptions remain. The Hippo-related pathway in ciliates was notably reported to contribute to the regulation of cilia biology as well as to the establishment of cell polarity (Tavares et al., 2012; Soares et al., 2019). However, there is no clear evidence that Hippo-related kinases in ciliates regulate cell cycle progression the way it was reported in other species such as yeast and denotes a certain degree of functional variability in this otherwise conserved pathway.

In comparison to the vast body of knowledge collected on the MEN-SPBs relationship in budding yeast or the SIN-SPBs in fission yeast, the precise contribution of human centrosomes to mitotic exit remains relatively unexplored. The Hippo signaling pathway is an important regulator of cell proliferation and apoptosis in higher eukaryotes. Given its importance in chromosome segregation and cytokinesis, the Hippo pathway is thus considered to play a functionally analogous role to the MEN (Hergovich and Hemmings, 2012). Although no clear Tem1 homolog has been identified in humans so far, Ras has been proposed to play a Tem1-like role in mitotic exit. Other MEN components located at SPBs appear to be conserved in humans, for instance centriolin, a centriole-appendage protein that transiently locates at the centrosomes. Thus, centriolin may play a similar role to that of Nud1 in promoting mitotic exit through its protein-protein interactions involving human MEN components (Pereira and Schiebel, 2001; Gromley et al., 2003). The centrosomes appear to act as a scaffolding structure for a broader range of regulators in humans, thus involving them in a multitude of intertwined pathways and cellular processes (Mayor et al., 1999; Chavali et al., 2014).

## 3 Beyond MTOC and Stoc Roles of Centrosomes/SPBs

The studies discussed above describe how SPBs/centrosomes act as essential signaling centers for many biological processes. However, multiple lines of evidence reveal the existence of additional non-canonical roles for centrosomes/SPBs. In this section, we describe how nature and evolution co-opt MTOCs into fulfilling roles that go beyond their typical contribution to cell shape, intra-cellular transport and cell division. These roles require MTOC activity in some cases but utilize microtubules in ways that exceed and/or diverge from their primordial function in eukaryotic cells.

### 3.1 SPB-Dependent Membrane Formation During Sporulation

**FIGURE 6:**
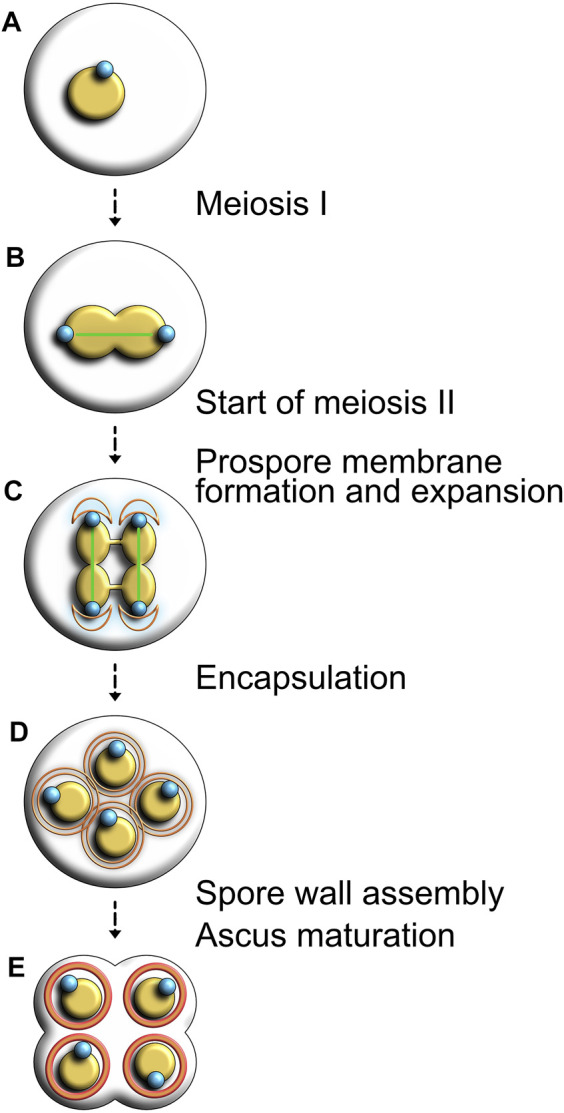
Visual representation of sporulation and ascus formation in budding yeast. **(A)** In response to environmental stressors, diploid yeast cells initiate the sporulation program. **(B)** Completion of meiosis I nuclear division. **(C)** After the second round of chromosome segregation, the prospore membrane (shown in orange) forms and expands around each duplicated SPB (shown in blue). **(D)** The membrane grows and encapsulates each haploid nucleus in the tetrad. **(E)** Spore wall assembly begins and the remnants of the mother cell breaks down.

When facing environmental stresses or severe nutrient deprivation, organisms ranging from bacteria and protozoa to plants and fungi can undergo sporulation as a way to adapt to environmental changes and increase the likelihood of their survival. Certain eukaryotic species, such as budding yeast, have the capacity to initiate sporulation as a form of specialized meiosis. This meiotic process allows for cells to shuffle and partition their genomic contents into different combinations, thus increasing the likelihood of progeny survival. In yeast, vegetative cells enter into premeiotic S phase. After completion of S phase, replicated DNA is partitioned into four haploid nuclei, which constitute the backbones of the four daughter cells to be created (Figures 6, steps A–C). Next, a membrane compartment, called the prospore membrane, matures and surrounds the four newly created daughter nuclei (Figure 6D). This step is vital for spore maturation as it gives rise to thick spore walls required for chromatin compaction and protection of cells from harsh environmental conditions (Roeder and Shaw, 1996; Coluccio et al., 2004; Suda et al., 2007; Neiman, 2011). Finally, the remnants of the parental cell collapse around the dormant progeny (the asci) to give rise to four mature haploid cells (Figure 6E) (Neiman, 2005).

SPBs support the initial construction of the prospore membrane, but the developmental reprogramming of vegetative cells that leads to sporulation alters their composition and function. During meiosis I, SPB duplication is similar to the process observed during mitotic division, but meiosis II induces multiple changes in SPB constitution that turns this organelle into a focal point for membrane formation. Most of its outer plaque components are replaced with specific proteins required for sporulation. During meiosis II, Mpc54, Spo74 and Spo21/Mpc70, three meiotic plaque components, act as substitutes for Spc72 on the cytoplasmic face of the SPBs. Instead of interacting with microtubules, Mpc54 and Spo21/Mpc70 cooperate with Nud1, Cnm67 and Spc42. The mechanistic process underpinning prospore membrane extension is not well understood, but we know that each prospore membrane surrounds its respective SPB in a semi-circular conformation prior to extension. Each membrane thus captures half of its corresponding nucleus, eventually forming walls englobing the entire nucleus (Neiman, 2011). In their research touching on prospore membrane formation, Knop and Strasser observed that levels of Mpc54 and Mpc70 peaked towards the end of meiosis II and plummeted shortly after, suggesting for a restricted role of these proteins exclusively in the formation of the meiotic plaque. Assembly of the prospore membrane was also shown to rely on Don1, a protein emerging towards the middle stages of meiosis I. Using immuno-electron microscopy, authors reported that Don1 localizes to the prospore wall during meiosis II and was proposed to be a marker for prospore membrane formation (Knop and Strasser, 2000). In a situation where meiotic SPB components are mutated or otherwise deficient, prospore membranes fail to engulf the four nuclei and the sporulation process collapses (Knop and Strasser, 2000), underscoring the essential nature of SPBs for this process.

### 3.2 MTOC as Linchpins for Cellular Reprogramming of Quiescent Cells

Eukaryotic cells rely heavily on stimuli provided by their immediate surroundings to make cell proliferation decisions. In situations where nutrients become limiting and proliferation is impossible, cells have the ability to initiate stress survival programs that enable them to better face environmental hazards. A cellular state termed quiescence can also be induced when nutrient become scarce or in the presence of specific developmental cues.

Quiescence is a common dormant state in wildlife (Gray et al., 2004; Zhang et al., 2019). Upon entering quiescence, cells temporarily halt their division cycle, thus allowing time for the surrounding environment to replenish its resources (Sagot and Laporte, 2019). This process, routinely observed in unicellular eukaryotes, is also common in multi-cellular organisms including humans, where the quiescent state preserves and maintains embryonic stem cell pools in adult tissues until actively needed for homeostasis or tissue repair (Cheung and Rando, 2013). In yeast, the decision to favor quiescence over other stress coping strategies can be determined by the availability and type of carbon source present in the environment. When ethanol is the predominant carbon source, sporulation and ascospores formation is the main stress coping strategy of budding yeast. Conversely, limited availability of a high-energy fermentable carbon source such as glucose makes quiescence the preferred route to maintain cellular homeostasis and redox equilibrium (Tomova et al., 2019).

**FIGURE 7:**
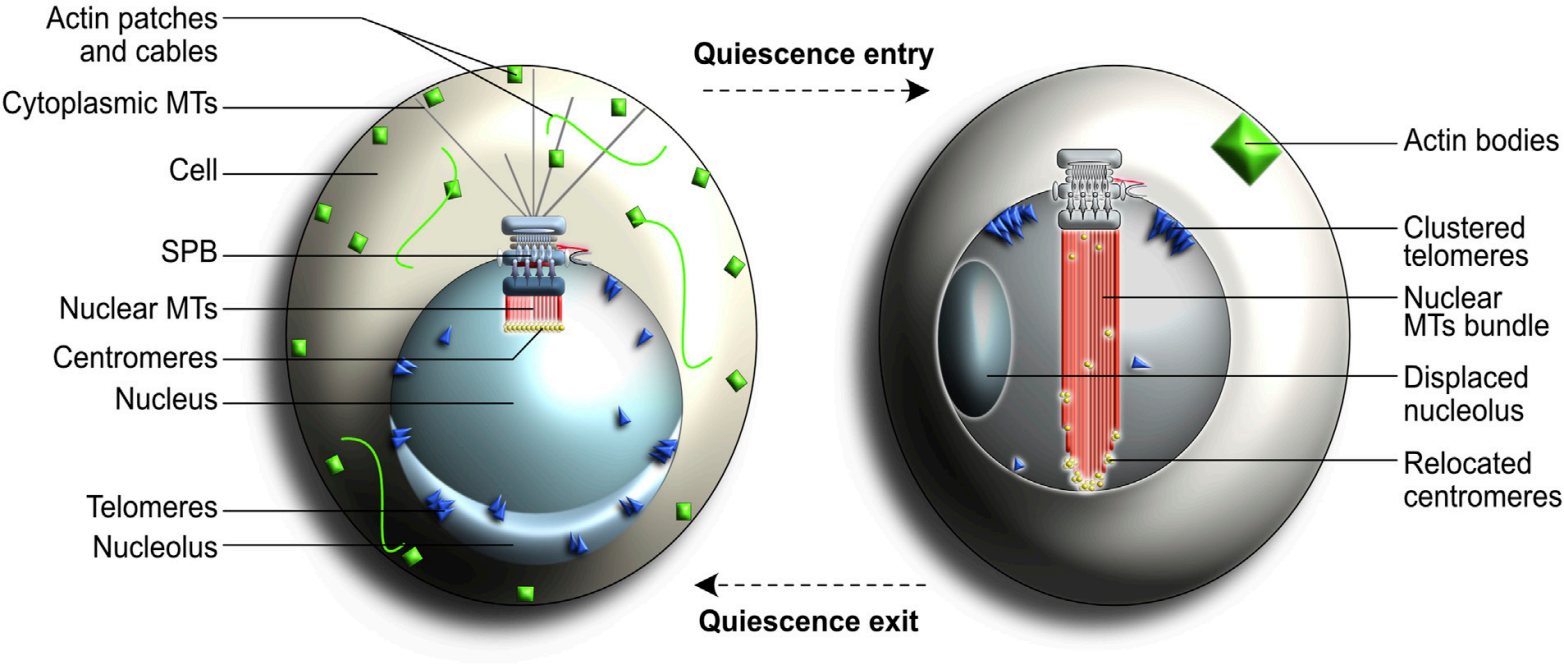
Cellular changes associated with the quiescent state in yeast. These changes include the disappearance of cytoplasmic microtubules (MTs) and formation of a nuclear bundle of MTs (nMTs) that spans the entire nucleus. Centromeres (shown in yellow) normally cluster together at the end of nuclear MTs in interphase cells (left) but get redistributed along the length of the newly formed nMT bundle in quiescent cells (right). Chromosome arms are omitted from this figure to simplify the representation. See text for more details.

Entry into quiescence induces major changes in cellular organization and physiology, including appearance of internal structures such as storage granules and actin bodies (Sagot et al., 2006; Narayanaswamy et al., 2009; Noree et al., 2010; Liu et al., 2012; Laporte et al., 2013; Shah et al., 2013; Sun and Gresham, 2020). The typical Rabl nuclear configuration, in which centromeres are clustered to one side of the nuclear envelope and concomitantly attached to the SPB, is replaced by a simplified nuclear arrangement in quiescent cells (Figure 7) (Guacci et al., 1997; Jin et al., 1998; O’Toole et al., 1999; Jin et al., 2000; Bystricky et al., 2004; Laporte et al., 2013). This response is fully reversible because quiescent cells typically revert back to the standard Rabl configuration in less than an hour after nutrients are replenished in their immediate environment. This rapid response to environmental cues is highly beneficial for most unicellular organisms and is thought to provide cells with increased competing fitness and enhanced survival chances (Laporte and Sagot, 2014).

An important quiescence hallmark in yeast is the assembly of a long and highly stable array of nuclear microtubules (nMTs) which spans the entire length of the nucleus and consequently displaces the nucleolus (Laporte et al., 2013; Laporte and Sagot, 2014). Chromosomes, which remain tightly attached to the SPB-generated microtubules, become spread along the length of the newly formed nMTs array. Whilst the exact purpose of this nuclear rearrangement during quiescence remains unclear, this selective chromosomal relocation has been proposed to influence gene transcription and mRNA export efficiency (Taddei and Gasser, 2012; Laporte and Sagot, 2014).

SPBs, that form the nMTs array in quiescent cells, are important executioners of the quiescence program. Accordingly, mutations that cause shifts in nMTs array length or stability impede quiescence-related nuclear reorganization and leads to quiescence defects, genomic instability and decreased likelihood of survival (Jin et al., 1998; Gray et al., 2004; Laporte and Sagot, 2014). Likewise, mutations affecting MT nucleation in SPB components, as well as in other organelles or transduction signal pathways involved in quiescence, may drastically reduce cell survival (Gray et al., 2004; Laporte and Sagot, 2014). *XRN1* (also known as *KEM1*) encodes an exonuclease involved in nutrient signaling. Mutated *xrn1* impaired relay of nutritional information to the SPBs, consistent with a possible role for the SPB as a signaling platform during quiescence (Werner-Washburne et al., 1993).

Although a few rare mutant cells survive and are capable of returning to a cycling state upon replenishment of environmental nutrients, the likelihood of survival of their offspring is greatly reduced; a surviving quiescence mutant will confer genomic instability to its progeny, often resulting in cell death (Laporte and Sagot, 2014).

It is unclear if centrosomes play a similar role as SPBs in mammalian cell quiescence. The formation of a nMT array is unlikely to occur in mammalian cells because centrosomes are typically not embedded in the nuclear membrane in higher eukaryotes. However, centrosomes may act as a key docking platform to regulate protein kinase A (PKA) signaling in the early stages of quiescence, as suggested by Gray et al. (2004). Furthermore, the process of quiescence has often been correlated with the formation of a primary cilium in mammals (Tucker et al., 1979; Laporte and Sagot, 2014). Given the requirement for cilium resorption in differentiated cells prior to cell division, the presence of a primary cilium in quiescent cells has been proposed to act as an important biological checkpoint. This theory would satisfactorily correlate with a cell’s need to assess the state of its external surroundings prior to reverting back to a cycling state (Kim and Tsiokas, 2011 as cited in; Laporte and Sagot, 2014). Further studies will be necessary to define more precisely the contribution of centrosomes to mammalian cell quiescence.

## 4 Closing Remarks

Centrosomes and SPBs are cellular organelles mainly recognized for their role as microtubule nucleators (MTOCs) crucial for cell shape determination, intra-cellular transport and cell division. While there is little debate that this viewpoint is well justified, the importance of centrosomes/SPBs in other cellular processes must not be overlooked. Indeed, these organelles also act as key players in the transduction of several signalization events and in the implementation of important differentiation programs. Through their roles as intracellular docking platforms that enhance kinase-substrate interactions, centrosomes/SPBs effectively function as important STOCs. This role is achieved through the regulated formation of supramolecular protein assemblies on the surface of MTOCs. The scale and compositional complexities of these assemblies suggest that STOCs provide a unique regulatory environment for signaling events. Moreover, the dynamic nature of their location/movements during the cell cycle suggest a capacity for decoding and translating spatio-temporal cues into transduction events. Overall, centrosomes/SPBs are indispensable to ensure cellular fitness and mutations in these organelles can lead to severe pathologies, ranging from microcephaly to cancer (Jaiswal and Singh, 2021). Given their versatile influence in cell proliferation and signaling events, future research efforts focused on the MTOC-independent roles of centrosomes could be a fruitful path for discovering therapeutic targets in the treatment of several diseases, including cancer.
